# Supplementary material for: Assessment of palladium concentration in drill cores using laser-induced breakdown spectroscopy (LIBS)
Source: PLoS One. 2025 May 19;20(5):e0320584. doi: 10.1371/journal.pone.0320584 (PMC12088053; doi:10.1371/journal.pone.0320584)
Supplement: S1 Table — (DOCX) [file pone.0320584.s001.docx]

**S1 Table.** Lac des Iles designation of the drill cores discussed in this work

| Core | Type | Zone | Sample number | Hole number | Depth range (m) |
| --- | --- | --- | --- | --- | --- |
| A | Gabbronorite | B3 | X097924 | 18-805 | 189-190 |
| B | Gabbronorite | B3 | A0126692 | 19-520 | 142-143 |
| C | Gabbronorite | B3 | A0126695 | 19-520 | 145-146 |
