# Supplementary material for: Assessment of palladium concentration in drill cores using laser-induced breakdown spectroscopy (LIBS)
Source: PLoS One. 2025 May 19;20(5):e0320584. doi: 10.1371/journal.pone.0320584 (PMC12088053; doi:10.1371/journal.pone.0320584)
Supplement: Attachment — Submitted filename: plos1.docx [file pone.0320584.s004.docx]

**Comments to the Authors**:

The manuscript reports measurements of palladium concentration in drill cores using Laser-Induced Breakdown Spectroscopy (LIBS). The subject matter is interesting and relevant, particularly in addressing the number of minimum measurements required for a reliable assessment of palladium concentration. However, the presentation lacks clarity, and several critical aspects of the manuscript require significant improvement before it can be considered for publication.

1. The rationale behind the choice of varying numbers of measurements for three different cores is not explained. It is crucial to justify these experimental parameters.

2. The inclusion of measurements showing a negative error in concentration estimation is highly questionable. In effect the cumulative negative errors result in nullifying a certain number of positive measurements. The details on why negative values are obtained and the rational for their inclusion is not clear. Also, it will be worth mentioning the percentage of these measurements.	How will the results change if only the positive values are considered.
3. The theoretical section is not clearly connected to the experimental results. The authors must explicitly state the purpose of this section, its relevance to the current study, and its potential implications for future experiments. How does this study enhance the understating, particularly for future measurements/Does it help in reduced no of measuserments? Also this section is too lengthy.
4. Figure 4 is not readable and does not effectively convey the results. It would be more appropriate to present the data in three separate graphs for clarity. Additionally, the mean concentration should be clearly indicated in each graph to enhance interpretability.
