# Supplementary material for: Assessment of palladium concentration in drill cores using laser-induced breakdown spectroscopy (LIBS)
Source: PLoS One. 2025 May 19;20(5):e0320584. doi: 10.1371/journal.pone.0320584 (PMC12088053; doi:10.1371/journal.pone.0320584)
Supplement: Attachment — Submitted filename: Response to Reviewers.docx [file pone.0320584.s006.docx]

**Reviewer #1: It is recommended to include LIBS spectra in this article, improving discussion based on the empirical evidence in which obtained through the LIBS method. The empirical data could be useful to support whereas the LIBS method is more effective than conventional methods regarding Pd analysis.**

We have incorporated a new Figure 2, which provides an example of a raw spectrum obtained at a specific position in core A. This figure also highlights the issue of noise in LIBS measurements, as clearly demonstrated in the displayed spectrum.

Regarding the efficiency of LIBS compared to conventional methods: as noted in the introduction, conventional techniques involve "a laborious, time-consuming, and energy-intensive sequence of grinding, homogenization, and dissolution, typically requiring more than a day." This estimate excludes the additional time needed for sample transport to often distant laboratories. In contrast, LIBS can rapidly scan the surface of a core on-site within minutes, with spectra processed in real time. However, as emphasized in the conclusion, LIBS is not intended to replace conventional methods where high accuracy is paramount. This limitation arises in part because LIBS is a surface analysis technique, whereas conventional methods analyze pulverized core samples, providing a more representative average composition.

**Reviewer #2: The manuscript reports measurements of palladium concentration in drill cores using Laser-Induced Breakdown Spectroscopy (LIBS). The subject matter is interesting and relevant, particularly in addressing the number of minimum measurements required for a reliable assessment of palladium concentration. However, the presentation lacks clarity, and several critical aspects of the manuscript require significant improvement before it can be considered for publication.**

**1. The rationale behind the choice of varying numbers of measurements for three different cores is not explained. It is crucial to justify these experimental parameters.**

The number of measurements taken for the three cores varied depending on their structural quality and specific circumstances. For example, the rounded section of core A was cut to create a third flat surface, ensuring a more representative sampling of the core. This modification was performed as part of the detailed study reported in Reference 10 (Selmani et al., 2022), but it was not repeated for cores B and C due to the time and complexity involved. Additionally, all cores were delivered in multiple fragments, some of which were broken at the sides. As a result, only smaller, rectangular laser shot arrays could be applied to certain fragments within our laboratory setup. This point is addressed in the revised manuscript.

**2. The inclusion of measurements showing a negative error in concentration estimation is highly questionable. In effect the cumulative negative errors result in nullifying a certain number of positive measurements. The details on why negative values are obtained and the rational for their inclusion is not clear. Also, it will be worth mentioning the percentage of these measurements. How will the results change if only the positive values are considered?**

We agree with the reviewer that the concept of negative concentrations is meaningless when taken out of context. Their inclusion is merely a mathematical twist that simplifies the statistical treatment in our data. As the reviewer noted, negative concentrations counterbalance the excess of positive concentrations caused by noise, thus providing a more balanced and accurate analysis.

A discussion of the new Figure 2 of the revised manuscript, which shows a spectrum with spectral lines and noise, aims to address this point in more detail.

As discussed in section 3.4 of Reference 10, 88% of the data collected for core A showed palladium concentrations below 10 ppm, which means that slightly less than half of them (41%) are negative (most of them being very close to 0), given the approximate symmetry of the distribution around the zero concentration. Despite this 88%, the total contribution of concentrations below 10 ppm (including negatives) accounts for only 2% of the core’s average concentration, with the remaining 98% derived from local concentrations exceeding 10 ppm. This reflects the nuggety nature of palladium distribution in the ore. Excluding negative concentrations would skew the results by only capturing the excess positive concentrations introduced by noise. Conversely, using a threshold of >10 ppm to filter out noise partially addresses this issue but is not universally applicable, as the threshold may vary across datasets. Adding all concentrations, including the negatives, provides a safer and more accurate method for calculating the average concentration. This approach ensures that noise effects are effectively neutralized without introducing arbitrary thresholds.

**3. The theoretical section is not clearly connected to the experimental results. The authors must explicitly state the purpose of this section, its relevance to the current study, and its potential implications for future experiments. How does this study enhance the understating, particularly for future measurements/Does it help in reduced no of measurements? Also this section is too lengthy.**

In our view, the theoretical sections (Sections 3.2 and 3.3) are the most compelling parts of the paper, as they provide a comprehensive discussion that extends beyond the limitations of the available experimental data. We believe their length is appropriate given their significance, and their motivation is clearly articulated at the outset of Section 3.2:

*"In this section we discuss the approach of using an analytical concentration distribution instead of a particular set of experimental data as in the previous section. This allows one to establish a conceptual framework without being restricted to a particular set of* $M$ *measurements, to clarify the uncertainties associated with a finite set of* $M$ *measurements, to understand the effects of noise in the measurements, and to compute probabilities for arbitrary mean concentrations* $\mu$*."*

This introduction provides the necessary context for the depth of analysis presented in these sections and answers the reviewer’s first question. Regarding the reviewer’s second question, the theoretical sections do not directly aim to reduce the number of measurements. Instead, they focus on optimizing the use of measurements by fostering a deeper understanding of potential sources of uncertainty. This allows for more informed interpretation and application of the experimental data, enhancing the reliability and robustness of the conclusions drawn. It also provides a model of the palladium distribution in the Lac des Iles ore.

**4. Figure 4 is not readable and does not effectively convey the results. It would be more appropriate to present the data in three separate graphs for clarity. Additionally, the mean concentration should be clearly indicated in each graph to enhance interpretability.**

Thank you for your advice. We have split the figure in question into three separate ones.
