# Supplementary material for: Assessment of palladium concentration in drill cores using laser-induced breakdown spectroscopy (LIBS)
Source: PLoS One. 2025 May 19;20(5):e0320584. doi: 10.1371/journal.pone.0320584 (PMC12088053; doi:10.1371/journal.pone.0320584)
Supplement: Attachment — Submitted filename: Response to Reviewers2.docx [file pone.0320584.s007.docx]

We thank the reviewers for taking the time to evaluate our manuscript and for sharing their concerns and recommendations.

At this point, only minor changes to the manuscript were recommended:

Reviewer #1: It will be better if the english style of the manuscript is improved, so that all scientific information and discussion based empherical data will be deliverd effectively

We are sorry that some parts of the paper seemed unclear to Reviewer #1. Although none of the authors are native English speakers, this is not a criticism we have typically received from reviewers of our other papers. Nevertheless, we have revised our manuscript using various software, including DeepL Write set to American English, and they recommended a few changes which will likely improve the English quality of the paper.

Reviewer #3: “Thanks for addressing my comments. I think the manuscript can be published after a few minor tweaks. At this point, just one revision is needed: please include the pictures of drilling samples. If you can, it would be great to see images of the sample surface before and after LIBS detection.”

We appreciate the idea of showing some photos of our work specimens, although more are shown in our previous publications. In the new Fig 2, we show photos of raw core fragments similar to those used in this work, and the laser-scanned surface of a fragment together with its mirror image obtained by trimming the round side of the fragment. Unfortunately, we did not have photos of the same surface before and after laser scanning.

We hope that this new version of our manuscript is to your satisfaction.
